# Measuring the world’s rivers with videos from Space

**DOI:** 10.1038/s41598-025-22413-4

**Published:** 2025-11-05

**Authors:** Nick Everard, Mark Randall, Guy Schumann, Sunita Sarkar, Harry Dixon, Ron Hagensieker, Alexander Dolokov

**Affiliations:** 1 UK Centre for Ecology and Hydrology, Wallingford, UK; 2 Queensland Government, Cairns, QLD Australia; 3 RSS-Hydro, Kayl, Luxembourg; 4 Osir.Io, Berlin, Germany; 5 wAIn, Berlin, Germany; 6 School of Geographical Sciences, University of Bristol, Bristol, UK

**Keywords:** Hydrology, Hydrometry, Velocimetry, Streamflow, Flood, Satellite, Environmental sciences, Hydrology, Natural hazards, Optics and photonics

## Abstract

The accurate measurement of volumes of water flowing in the world’s rivers is of critical importance for people, for nature and for industry. Our planet’s rapidly changing climate is increasing this need, as water becomes scarcer as a resource and more dangerous as a hazard. Additionally, many river monitoring networks globally are inadequate and declining. To date, satellite-based methods used observations of river width, water surface height, and water surface area, but did not include the critical parameter of water flow speed. Here, we present a significant advance by demonstrating a method for determining water flow speed with a high degree of accuracy using video imagery obtained by a constellation of low-earth-orbit satellites. The very high resolution of the video imagery also allows observations to be made in rivers as narrow as 70 m wide. Through a programme of ground-based validation measurements, we have demonstrated agreement in discharge measurements better than 5% at a range of river sites around the world. This development can herald a step change in capabilities for the measurement of rivers globally, allowing observations in remote locations, and during extreme events such as floods, with no need for people or equipment to be on site.

## Introduction

Accurately quantifying the volumes of water carried by the world’s river systems is of great importance for human activities, for maintaining natural ecosystems and for managing hazards relating to fluvial flood risk^1–3^. The triple challenges of growing human populations, increasing urbanisation and unpredictable weather extremes driven by climate change^4^ amplify the need for accurate and timely river flow information globally^5^. Flooding is the costliest of natural disasters, with the global average annual loss associated with river flooding estimated at $110 bn in 2018^6^. Millions of people are exposed to floods worldwide, and impacts are expected to increase threefold by 2050^7,8^.

Accurate measurement of streamflow is however a complex, costly, and potentially hazardous challenge to undertake instrumentally^9^, requiring significant investments of equipment and people time^10^. Hydrological modelling can be an effective tool for quantifying water availability and hazards but is often limited by available input data and poor regional representativeness, resulting in inconsistent results^11^. Ongoing pressure on the budgets of national hydrometric monitoring services around the world underlines the need for new cost-effective, low infrastructure monitoring solutions^12^.

In this context, developing novel methods for measuring river flow is an urgent imperative^13^. Satellite-based remote sensing of rivers has shown considerable potential in recent decades^14^, but for direct observations of streamflow it has been constrained by factors such as the availability and resolution of sensing technologies, and perhaps most significantly, the difficulty of observing water flow speed in open channels. To improve results, various approaches have been tried that integrate hydraulic theory into calculations, for example developing hydraulic models based upon remotely sensed observations of river width, or water surface elevation^15,16^. Another major consideration for operational and, in particular, extreme event monitoring is the revisit interval for the satellites upon which these studies were based, which can exceed 35 days^17,18^.

Launched in December 2022, the NASA/CNES Surface Water and Ocean Topography (SWOT) mission advances satellite capabilities for hydrology considerably with a wide-swath radar altimeter that can penetrate clouds and can enable improved observations for both marine and inland water. The derivation of river discharge is a specific objective for the SWOT mission, but this will be constrained in its applicability by limitations such as spatial scale (discharge is calculated for 10 km river reaches and only for rivers in excess of 100 m wide) and the long delay between observations with (typically) just two observations in each 21-day orbital cycle possible^19^.

The SkySat constellation^20^ operated by commercial satellite company Planet Labs PBC is one of the few constellations offering the optical video imagery that is essential for our water speed analysis methods. The 21 SkySat satellites operate at orbital altitudes of 400 and 450 km and provide panchromatic still and video imagery for user-defined acquisitions. The large number of satellites and flexible targeting of Areas Of Interest (AOIs) enable Planet to claim anything up to 12 observations a day of a given location with the SkySat constellation, offering great potential for events such as rapidly developing flooding disasters.

Research such as that published by Legleiter and Kinzel^21^ and Masafu et al.^22^ demonstrates that the velocity of water in rivers can be observed using optical satellite video imagery and particle imaging velocimetry without the aid of, for example, ice tracers^23,24^. The research presented here advances upon these works by employing a more robust Space Time Imaging Velocimetry (STIV)^25,26^ velocity determination algorithm, by including direct comparisons with both ADCP and drone derived velocity and discharge values, and by assessing results across a range of rivers. Our analysis was based upon very high-resolution satellite video imagery, with the potential for sub-daily revisit times. We applied a newly developed perspective-transformation based algorithm to ensure that the flow of the water could be identified without any bias from instability in the imagery and analysed the resultant imagery with well-proven methods for deriving water surface flow speeds from low-cost aerial camera drones^27^. We validated the results against field-derived data across multiple sites (see Table 1) and tested the method on a major flood event, with a series of videos of a location on the Indus River in the Khyber Pakhtunkhwa province, 80 km below the Tarbela Dam during the 2022 flood disaster. We named the method presented here ‘FluViSat’ to represent the application to fluvial observations of videos obtained by satellites.

**Table 1:** Sites used for validation of satellite observations.

Country	Site name	Site type	Channel width (m)	Latitude, longitude
Australia	Burdekin river at Hydrosite	River	180–200	−20.628567, 147.165902
UK	Falls Of Lora, Connel	Tidal	150–300	56.456156, −5.393815
UK	River Tweed at Norham	River	70	55.722825, −2.162671
Japan	Uono River at Negoya Bridge	River	130	37.2458506, 138.9275143
Switzerland	River Rhine at Rhine Falls	River	100	47.674231, 8.609999

## Results

A total of 20 SkySat videos were acquired for the FluViSat project. For eight of these, field-based observations allowed that the satellite-derived surface water velocity and river discharge be compared to reference values obtained in the field. These eight observations form the bulk of the analysis and discussion in this article. A further six videos, acquired to demonstrate the potential of the method for observing a major flood event on the Indus River in Pakistan, are also discussed. Whilst no on-site validation was possible for these acquisitions, the fact that six high-quality videos were obtained and that velocities were able to be derived for several locations within each video gave us some opportunity to compare and gain confidence in the results. The remaining six videos were single acquisitions obtained at a range of locations, mostly coincident with significant flood events. Whilst velocimetry processing was carried out on the data from these sites, with generally very good-looking results, project funding and resources did not permit more detailed analysis for inclusion in this manuscript.

For the eight videos where on-site validation was possible, extremely good results were achieved, with satellite derived velocity and discharge values deviating by ^+^/- 5% from ADCP and drone-derived values (see Table 2; Table 3). These results are all well within our target of 10% and represent an excellent result for any form of river discharge measurement.

**Table 2:** Comparison of width-averaged surface velocities derived from satellite videos, ADCPs and aerial drones.

Site	Date	Satellite surface velocity (m/s)	Field data surface velocity (m/s)		Average field-derived surface velocity (m/s)	Difference satellite vs field (%)
			ADCP*	**Drone**		
Burdekin River at Hydrosite	7/2/22	1.671	1.685	1.667	1.676	−0.30
Burdekin River at Hydrosite	8/2/22	1.585	1.602	1.585	1.594	−0.56
Burdekin River at Hydrosite	18/5/22	1.677	-	1.622	1.622	3.39
Uono at Negoya Bridge	10/5/22	1.331	1.326^+^	1.331	1.329	0.15
Falls of Lora at Connel	18/3/22	1.758	-	1.676^++^	1.676	4.89
Falls of Lora at Connel	13/8/22	1.599	-	1.648^+++^	1.648	−2.97

*Acoustic doppler current profiler.

+ ADCP data was collected 1 km upstream of the satellite data.

+ + Drone data from July 16th, 2022.

+ + + Drone data from July 15th, 2022.

**Table 3:** Comparison of calculated discharge derived from satellite videos, ADCPs, aerial drones and gauging stations.

Site	Date	Satellite derived discharge(m^3^/s)	Field data discharge (m^3^/s)		Field derived discharge (m^3^/s)		Difference satellite vs field (%)
			ADCP*	**Drone**	**Average Drone & ADCP**	**Gauging Station**	
Burdekin River at Hydrosite	7/2/22	1040	1042	1038	1040	-	−0.05
Burdekin River at Hydrosite	8/2/22	900	915	900	908	-	−0.82
Burdekin River at Hydrosite	18/5/22	1697	-	1688	1688	-	+ 0.57
Uono at Negoya Bridge	10/5/22	189	182	191	186.5	-	+ 1.53
River Tweed at Norham	17/3/22	113	117^+^	116^+^	116.5	110.5	−2.58
Rhine at Rhine Falls	17/10/22	324.3	-	-	-	309	+ 5

*Acoustic doppler current profiler.

+ Drone and ADCP data from 15^th^ March 2022.

**Fig. 1:**
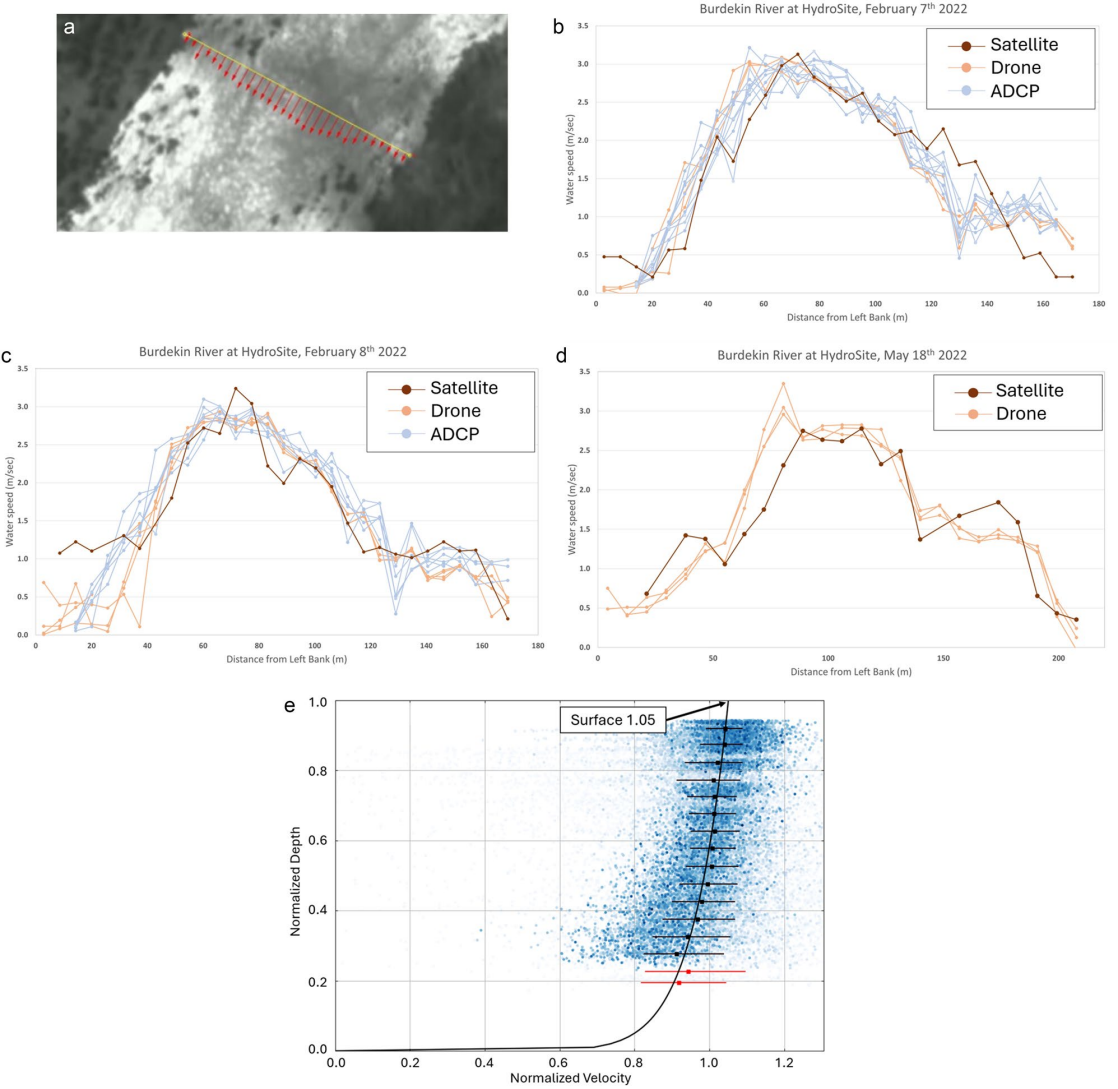
Satellite and field derived results for the Burdekin River, Queensland, Australia during high flows in 2022. (**a**) Velocity vectors derived from a SkySat satellite video from the Burdekin River, Queensland, Australia, on February 7^th^, 2022. Modified (cropped) frame from satellite video imagery obtained by Planet Labs’ SkySat S104 satellite, reproduced with kind permission of Planet Labs PBC. Processed in Hydrosoken HydroSTIV velocimetry software, version 1.2.2 (now superseded) https://www.hydrosoken.co.jp/en/hydrostiv/. (**b**) Comparison of water speeds derived from satellite, drone and ADCP for the Burdekin River, February 7^th^ 2022. The drone derived values represent surface water speeds, and so are directly comparable with the satellite results. The ADCP results represent the velocity reading that is obtained closest to the water’s surface, at a depth of 0.34 m. (**c**) As with (**b**), but for the same location on February 8^th^ 2022. (**d**) Comparison of water speeds for the Burdekin River on May 18^th^ 2022. River conditions prevented ADCP deployment, so comparison made with drone readings only. (**e**) Normalised vertical velocity profile plot extracted from the ADCP measurement at Burdekin River Hydrosite on 7th February 2022, showing the relative magnitude of downstream velocity from the bed to the surface averaged across the full width of the river. The X axis represents a dimensionless water velocity scale with 1.0 representing the mean velocity in the vertical. The Y axis represents a dimensionless water depth scale, with zero being the riverbed and 1.0 being the surface. The black squares show the median values of normalized velocity at the normalized depth and the black whiskers on the squares show the range of 50 percent of the data in that median. Each small dot represents an ADCP velocity reading. The curve fitted through the dots is used in ADCP processing to calculate discharge in the unmeasured top and bottom sections where no data points (dots) exist. For surface velocity discharge measurements, the point at which this curve intersects the surface (the top of the plot area) informs the derivation of the surface alpha coefficient. The variability of near-surface data points is illustrated by the spread of the light blue points. The red points close to the bottom have been screened out as they are judged to contain insufficient data to contribute reliably to the development of the velocity profile curve.

High flows experienced in the Burdekin River in Queensland, Australia in February and May 2022, resulted in flow conditions well suited to optical velocimetry techniques (see Fig. 1a). By the time that the flood peak reached the validation site, the rain-bearing clouds had moved on, leaving the sky clear for satellite video acquisitions. Across three observations on the two flood events, satellite derived discharge calculations were within 1% of reference values obtained with an ADCP and an aerial drone (Table 2; Table 3; Fig. 1). These excellent results in part reflect the fact that, for the February measurements, ADCP data was obtained concurrently with the satellite imagery, meaning the river cross-section and vertical velocity profile information used was wholly representative of conditions on the day.

In May, a larger flood flow on the Burdekin made deployment of the crewed ADCP boat unsafe, and so comparisons were made solely with the aerial drone, using the river cross-section and discharge profile obtained in the earlier field trip for discharge calculations (Fig. 1). Despite these practical issues, the satellite derived results were still within 5% of the field results (Table 2; Table 3).

**Fig. 2:**
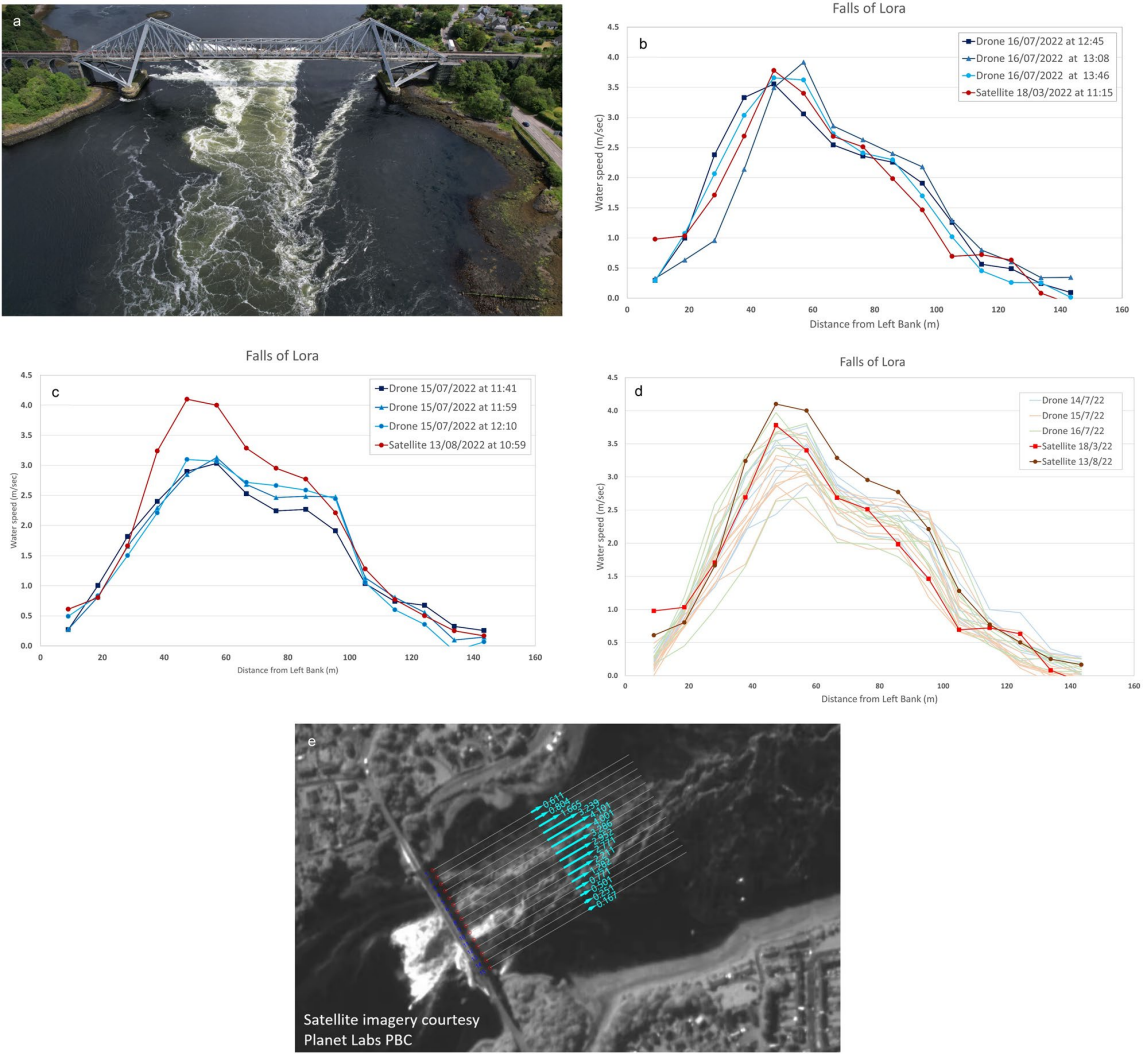
Satellite and field derived results for the tidal site, Fall of Lora at Connell, Scotland for various dates in 2022. (**a**) Aerial picture of the ebb tide at The Falls of Lora at 13:25 BST July 15^th^, 2022, showing the large area of complex flow. Picture taken with the drone that was used for velocimetry. The currents were too strong to allow the safe deployment of the ADCP. (**b**) Comparison of surface velocities for March 18^th^, 2022, satellite video, with tidal range of 2.79 m and three drone videos representing the same point in a tidal cycle of very similar magnitude on July 16^th^, with tidal range of 2.78 m. (**c**) Comparison of surface velocities for August 13^th^, 2022, satellite video, with tidal range of 2.98 m and three drone videos representing the same point in a tidal cycle on July 15^th^, with tidal range of 2.84 m. It appears from the velocimetry results, and from a timelapse camera deployed on the bridge that the tidal current on August 13^th^ was significantly stronger than for the comparison observations on July 15^th^. (**d**) Surface water speeds derived from satellite and drone-based velocimetry, where tidal ranges for drone observations were as follows: July 14^th^ 2.76 m, July 15^th^ 2.84 m, July 16^th^ 2.78 m; and tidal ranges for satellite observations were, March 18^th^ 2.79 m & August 13^th^ 2.98 m. (**e**) Velocity vectors at The Falls of Lora derived from SkySat video on August 13^th^, 2022. Modified (cropped) satellite imagery courtesy of Planet Labs PBC. Processed in Hydrosoken HydroSTIV velocimetry software.

At the tidal Falls of Lora at Connel, Scotland, the strength of the current made deployment of the ADCP sensor unsafe, which prevented the measurement of a cross-section and velocity profile, and hence the calculation of discharge (Fig. 2a). Instead, a comparison was made between surface velocities derived from the satellite videos and from the aerial drone. These comparisons were complicated by the fact that, due to cloud cover, it was not possible to obtain a satellite video concurrently with the drone measurements. An additional challenge was that during the cycle of the ebb tide, the magnitude and spatial distribution of flow through the constriction beneath the bridge varies considerably over a relatively short timeframe. To address these issues, a series of drone videos were obtained at intervals of around 20 min over the course of three ebb tide events of a similar magnitude to those for which satellite imagery was obtained. These were processed to provide a time-series of velocity distribution throughout the cycle of the ebb tides. Water speed results from the satellite videos were then compared to those that were closest to the same point in the cycle of a similar magnitude of ebb tide. With this approach, the average water speed calculated from the satellite imagery was found to be within 5% of that observed with the drone (Fig. 2b- e**; **Table 2), and the spatial distribution of water speeds matched very closely**.**

At two sites, challenges with acquiring satellite imagery required further processing and alternative means to validate results. At Uono River in Japan, the satellite did not lock on to the AOI and only a short length of the river was visible in the video. This required additional image processing, to isolate the frames where the river was in view and to stabilise the relatively rapidly moving field of view. This processing was managed by collaborators in Japan. Results derived from the stabilised satellite imagery are within 1.5% of the reference values obtained with an ADCP and a drone (Table 2; Table 3). At Rhine Falls in Switzerland, due to cloudy conditions, the satellite video could not be acquired until several weeks after the field trip to collect drone and ADCP data in August 2022. In addition, challenges in rectifying the imagery in a steep-sided wooded valley, meant the pre-processed video remained somewhat unstable, with some 14 m of downstream shift of the imagery apparent through the duration of the video. For the 30-s video, this translates into a low bias in water speed of 0.47 m/s. Since the erroneous downstream displacement of imagery was uniform across the width of the river, a velocity correction of 0.47 m/s was applied to all calculated water speeds to account for the instability in the imagery. Discharge was then calculated using the cross-section information from the August ADCP deployment, with an appropriate adjustment made for the increased water surface height. The calculated discharge was within 5% of the value reported by the Rhein Neuhausen Flurlingerbrücke river flow gauging station, the performance of which was confirmed during the earlier field trip (Table 3) Because of the long hiatus and associated change in river discharge, between the field and satellite data acquisitions and the issues with instability in the satellite video, a direct comparison of water speeds from drone and satellite sources is not provided in this paper.

Cloud cover prevented a satellite acquisition on the same day as the drone and ADCP measurements on the River Tweed at Norham. However, a satellite video suitable for processing was acquired two days after the field measurements were made, and with river discharge reported by Norham Gauging Station just 3% lower. A consequence of relatively low flow conditions prevailing at the time was that trackable features on the water’s surface at the location where the drone and ADCP data was collected were too scarce to yield usable results when the satellite video was obtained. However, because the footprint of the satellite video extended along a 1.8 km reach of the river, it proved possible to derive velocity readings at a location some 500 m further upstream, where a bend in the river was generating additional surface roughness features that could be tracked by the STIV software. In order to enable the calculation of discharge, the bathymetry of this reach of river was mapped at a later date with an ADCP and remotely operated survey boat. Using a cross-section derived from this survey and with a suitable adjustment for changes in water surface height, the satellite-derived discharge was within 3% of the reference value (Table 3).

## Extreme event use case

**Fig. 3:**
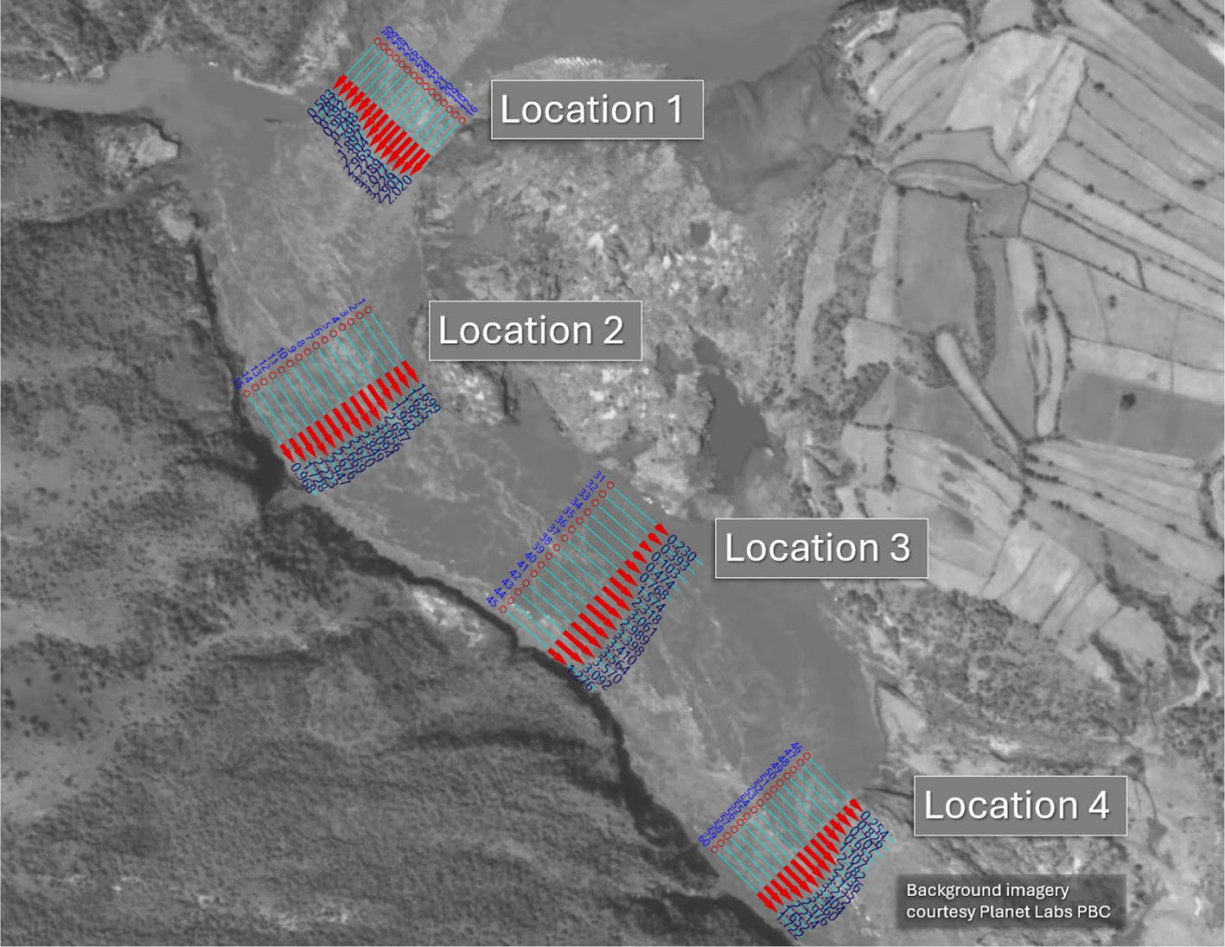
Velocity vectors for flood event in the Indus River, Pakistan. The four locations along the river where satellite velocimetry was derived for the Indus River on September 2^nd^ 2022, during the historic flooding that lasted from June to October of that year. Note how the magnitude of the red velocity vectors matches the flow patterns discernible in the imagery. Modified (cropped) frame from satellite video imagery obtained by Planet Labs’ SkySat S110 satellite, reproduced with kind permission of Planet Labs PBC. Processed in Hydrosoken HydroSTIV velocimetry software, version 1.2.2 (now superseded) https://www.hydrosoken.co.jp/en/hydrostiv/.

A potentially extremely valuable application of satellite streamflow observations can be to provide near-real-time surveillance of high-impact flood events globally. When disastrous flooding hit Pakistan in the summer of 2022, a series of six SkySat videos was acquired, between 1^st^ and 16^th^ of September, for the Namal Payan location on the Indus River in the Khyber Pakhtunkhwa province, 80 km below the Tarbela Dam. The location chosen for the satellite acquisitions displayed significant surface roughness features throughout the reach of river imaged by the satellite, which enabled velocimetry analysis at between three and four separate locations for each of the six videos acquired (Table 4; Fig. 3).

**Table 4:** A summary of observations for Namal Payan on the Indus River, Pakistan. Due to variations in satellite targeting of the AOI, not all locations were visible in every video, which is why not all the locations have the same range of dates.

Date videos acquired	Location 1	Location 2	Location 3	Location 4
1/9/22	1.79	1.26	1.33	
2/9/22	1.98	2.18	2.21	2.04
3/9/22	1.90	1.87	1.74	
6/9/22	0.80	1.34	1.79	
14/9/22		2.29	2.19	2.46
16/9/22			1.37	1.35
**Range of surface velocities derived from imagery (mean for location in m/s)**	0.80–1.98	1.26–2.29	1.33–2.21	1.35–2.46

Field validation was not possible at this site, and no other hydrometric data was available for comparison purposes. However, since velocimetry analysis was performed independently at between three and four locations within the frame of the videos acquired, a comparison of the magnitude and distribution of the calculated water speeds was possible. As can be seen in Fig. 3a, the magnitude and distribution of the calculated velocities are in very good agreement across the four locations and conform with the hydraulics of the site.

## Discussion

This project advances remote sensing of rivers considerably by demonstrating that the speed of flow of water on the earth’s surface can be accurately quantified through the analysis of very high-resolution satellite video imagery. The basic velocimetry methodology used is already well proven^28^, with the satellite bringing the benefit of global reach and a rapid response capability.

This study is distinguished from the work of Legleiter and Kinzel^21^ and Masafu et al^22^ who also explored the potential of satellite video for streamflow measurements, in a) analysing a larger number of videos from a range of locations, b) comparing satellite-derived velocimetry with simultaneously acquired drone-based velocimetry and c) in utilising the very robust Space Time Imaging Velocimetry software rather than particle imaging velocimetry techniques. By obtaining satellite videos coincident with field validation measurements for eight satellite acquisitions at five locations, we were able to demonstrate the success of the method across a range of waterbody types and flow conditions. The six acquisitions obtained during the flood on the Indus River, although unverified through field measurements, provided additional visually compelling evidence of the method’s potential. By comparing satellite-derived surface velocimetry results with surface velocimetry results obtained simultaneously with aerial drones, as opposed to depth-averaged ADCP data (Legleiter and Kinzel^21^) and a hydraulic model (Masafu et al^22^) we were able to undertake rather more directly comparable analysis.

Although the HydroSTIV software employed here is only able to resolve 1D (i.e. downstream) velocity vectors, the STIV analysis technique can provide a more robust and accurate method for reporting this, the critical variable required for river discharge measurements^29^. This robustness is particularly important when the features being tracked lack clarity in the imagery, as was sometimes the case with satellite videos.

Comparison with velocities derived from aerial drone and an ADCP at the Burdekin River site gives R^2^ values ranging from 0.73 to 0.82 (Table 5), which compare favourably with those achieved by Legleiter and Kinzel^21^ (0.34—0.39) and Masafu et al^22^ (0.32–0.52).

**Table 5:** R-squared values for observations on the Burdekin River at the Hydro site.

Regression analysis, Burdekin River	Satellite vs Drone	Satellite vs ADCP
February 7th 2022	0.756	0.810
February 8th 2022	0.817	0.734
May 18th 2022	0.821	-

Within the time and budgetary constraints of this proof-of-concept project, it was not possible to obtain multiple video acquisitions at any individual established river gauge aside from Hydrosite on the Burdekin River, where three observations were made. Two velocimetry comparison exercises were also undertaken at the Falls of Lora tidal site. The need to pay for the video acquisitions and decision to undertake direct comparisons of aerial video velocimetry with drones in a range of settings prevented a bigger programme of observations at any given location. Future work involving regular video acquisitions at or close to an established river gauging station could help further demonstrate the potential and limitations of the technique.

This project has demonstrated that satellite video-based measurements could potentially deliver valuable information to aid the observation and management of water in both routine operational and extreme event situations. For rapid-onset flood situations, the 21 satellites in the SkySat constellation can permit highly responsive acquisitions, and multiple observations of individual events, although work would be required to refine workflows to deliver results close to real-time. During the project, SkySat videos were generally shared with the FluViSat team within around 24–48 h of acquisition. The stabilisation and rectification procedures then added a day. The velocimetry analysis and calculation of discharge was a manual process taking around 90 min. To deliver a timely, reliable and effective solution with widespread applicability to monitoring requirements (including flooding) therefore would require a streamlined video delivery, pre-processing and discharge calculation workflow. This should be possible through the development of automated video processing and discharge calculation techniques. The supply of pre-processed (stabilised, rectified and optically optimised) video products from satellite operators could also dramatically improve responsiveness and perhaps lead to greater demand, including from other sectors. Additional requirements to enable an operationally effective service would include budgets to pay for tasking and agreed prioritisation of satellite acquisitions for hydrology.

Using the FluViSat method and a multi-satellite constellation such as Planet’s SkySat network, observations can potentially be made multiple times in a single day, at even the most inaccessible locations. With no need for people or equipment to be present in the field at the time of the observation, operational costs and risks to the safety of personnel and monitoring infrastructure are greatly reduced. River cross-sections obtained before or after a satellite video acquisition, or maintained in a database, can be used to enable the calculation of discharge, although consideration must be given to the stability of the river’s cross-section in particular in times of major flooding.

Further research is planned to build upon rapid advances in satellite remote sensing capabilities and to expand the range of sites and conditions in which satellite-based streamflow observations can be made.

### Insights and challenges

An unexpected finding was the degree to which variations in the satellites’ viewing angle affected the visibility of water surface features through the duration of satellite video clips. The satellites in the SkySat constellation were designed with attitude-control systems onboard that enable them to slew in order to obtain imagery both at nadir and off-nadir angles^30^. In simple language terms, the satellites can look ahead, behind or to the side to maximise their ability to obtain imagery of required locations. These attitude control systems are also employed to enable the satellite to ‘lock on’ to an area of interest (AOI) while acquiring the 30-s videos used in this project. This means that, as the satellite approaches a tasked AOI, attitude controls allow the satellite to start to look ahead and if necessary to the side of its current nadir view to begin imaging the AOI.

**Fig. 4:**
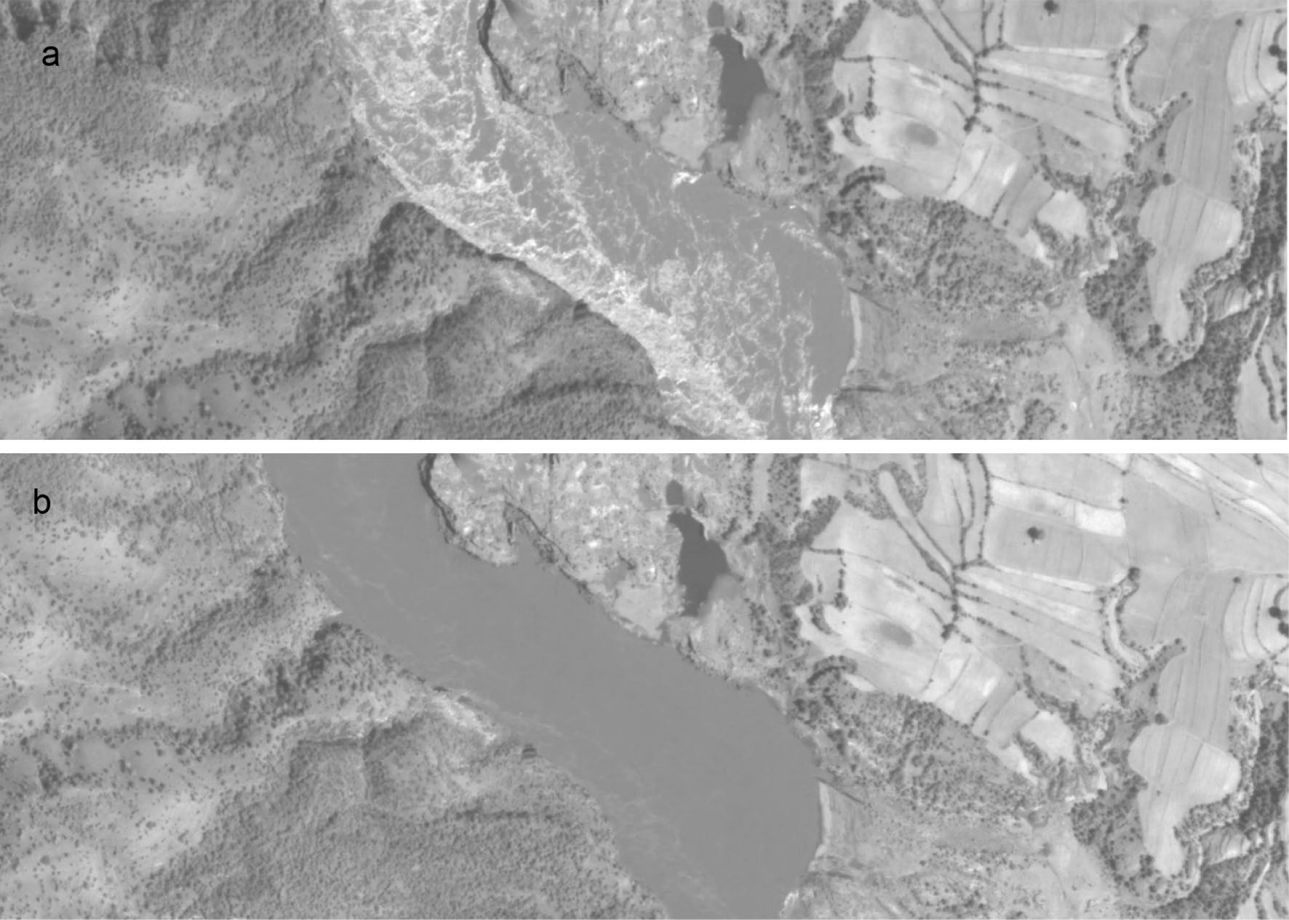
Variations in visibility of water surface features of SkySat videos Indus River, Pakistan, 14^th^ September 2022, showing (**a**) the start, and (**b**) the end of the SkySat video. Unmodified frames from satellite video imagery obtained by Planet Labs’ SkySat S112 satellite, reproduced with kind permission of Planet Labs PBC.

Depending on which SkySat in the constellation obtains the video and at what time of day, the viewing angle can vary considerably for any given AOI and will always change throughout the duration of the video, as the satellite approaches and then passes the AOI. For observing generally non-reflective land or built environment surfaces and features, this is usually not a problem. Due to the highly reflective nature of the water’s surface, a number of SkySat videos analysed showed a considerable variation in the brightness and clarity of water surface features, while illumination of the land surface barely changed at all, as illustrated in Fig. 4. For videos acquired in the northern hemisphere, the first 5–15 s of the video tended to have the clearest visibility of water surface features. Given the relatively small set of videos available for study, it is not clear at this stage if this is simply coincidence, or if there are factors that dictate this. Since this phenomenon can have a major impact on the suitability of satellite videos for velocimetry analysis, this is seen as a priority area for further research to develop a software tool that leverages the multiple daily acquisition times and azimuths permitted by the 21 satellites in Planet’s SkySat constellation to optimise the visibility of water surface features.

Cloud cover was the biggest challenge and is a common problem for optical satellite remote sensing methods. Issues with cloud cover impacted efforts to synchronize satellite and ground-based observations, restricted the usability of some videos, and prevented any satellite acquisition for certain target sites. A notable benefit of basing the research on the SkySat constellation, was that the 21 satellites allowed several acquisition attempts each day, greatly increasing opportunities to obtain cloud-free imagery. Future research will examine the potential of determining river flow speed with satellite sensors that are unaffected by cloud cover.

Very precise stabilisation and rectification of the video imagery proved to be critical to success, with any residual instability or distortion after processing potentially leading to errors in calculated water speeds, the impact of which would be greatest in slow moving water. This currently limits the method to water which is at least moderately fast flowing (in excess of 1 m/s) or has larger scale visible tracers such as rafts of foam, which can occur downstream of waterfalls and flow control structures, such as is the case at Rhine Falls in Switzerland. Future improvements to the optical quality and stability of satellite acquisitions, combined with better designed video tasking to optimise illumination are expected to extend the range of conditions in which the method is applicable.

As the SkySat constellation captures imagery in response to demand from customers, competition for satellite time can prevent acquisitions over certain locations at certain times where conflicting demands occur. Whilst we did not have a direct view of tasking demands and priorities, we were advised by technical staff at Planet Labs that this was the case for certain parts of the UK, Europe and parts of North America.

## Conclusions

The measurement of flow rates in rivers is essential for the effective management of water as a resource, and as a potential hazard. Monitoring using traditional methods is costly, sometimes dangerous, and monitoring networks on many major rivers are both inadequate and declining.

We have demonstrated that the speed of flow of water in the world’s rivers can be measured using high resolution videos acquired by low earth orbit satellites. We have also demonstrated that where river conditions are suited to the method, surface water speeds can be calculated for rivers less than 100 m wide, and with an accuracy comparable to that of many established methods^31^. Where good quality information exists on the river’s cross-section and vertical velocity profile, river discharge can be calculated with an accuracy of 5%, which is comparable to established techniques.

The ability to measure river flow speed directly and with rapid revisit times, can greatly improve the accuracy and utility of river discharge observations, improve models and represent a major advance for global hydrology and natural hazard management.

## Methods

### Overall workflow

**Fig. 5:**
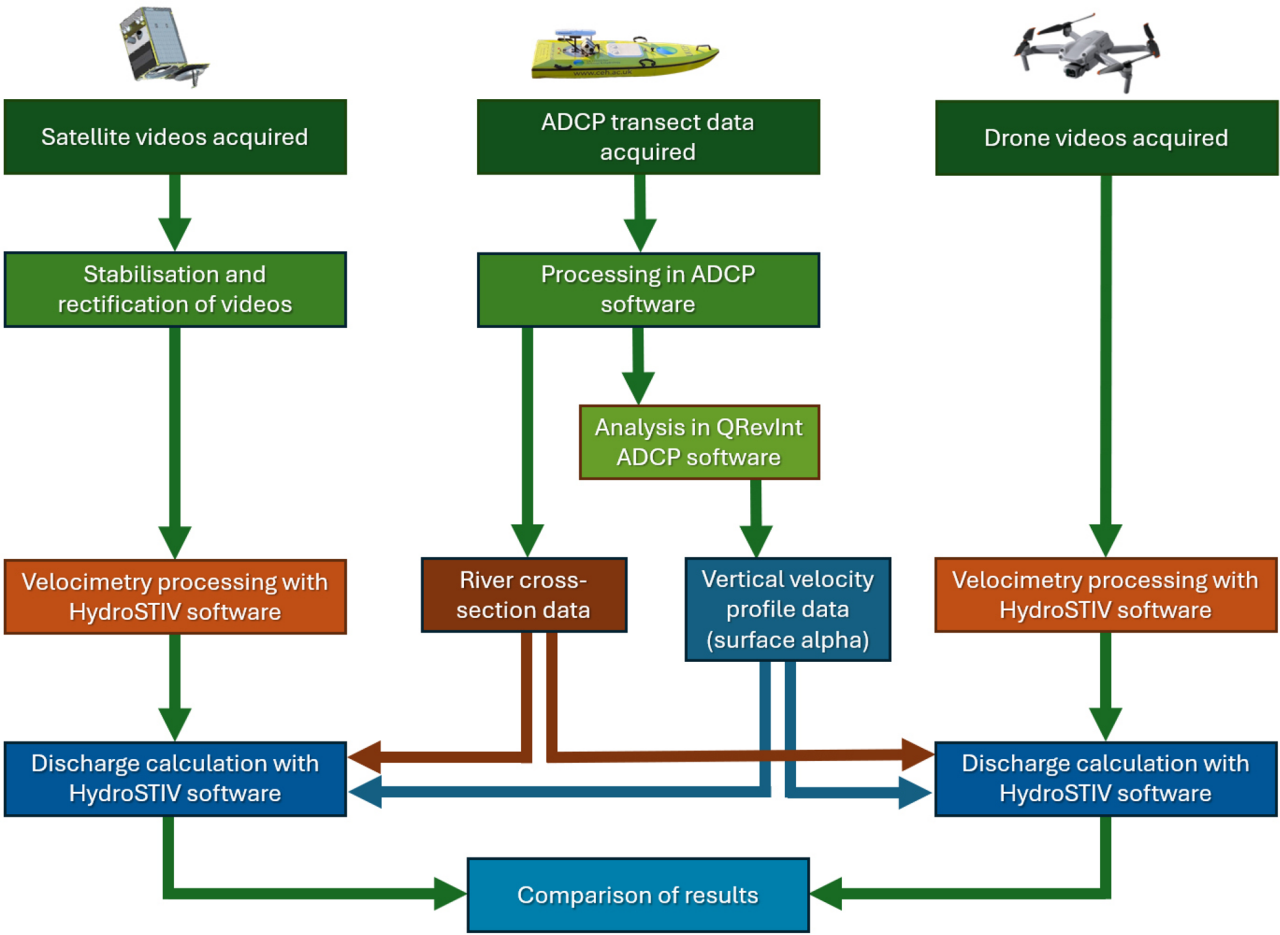
Velocimetry and discharge calculation workflow. The workflows employed for velocimetry and discharge calculation, showing how the ADCP measurements were used to provide cross-section and velocity profile data to enable discharge calculations for drone and satellite observations.

For the calculation of discharge, a standard velocity-area method was applied^32^. The workflow (Fig. 5) involved the acquisition and analysis of satellite and drone videos along with river discharge data obtained with an ADCP acoustic river flow sensor. Pre-processing of the satellite imagery was required to stabilise it and rectify it before velocimetry analysis. River cross-section and velocity profile information was then extracted from the ADCP datasets to enable the calculation of discharge from the drone and satellite data. By combining the surface velocimetry results with the ADCP derived variables, discharge could be calculated.

### Satellite earth observation video imagery data

The SkySat satellites can resolve features on terrestrial water surfaces smaller than a meter across and can shoot grayscale video clips up to 90 s in duration at 30 frames per second and with a resolution of 2560 × 1080 pixels^29^. Videos of 30-s in duration were acquired for this project.

Optically, the SkySat satellites feature an array of three identical 5.5-megapixel imaging sensors, and a 3.6-m focal length Cassegrain telescope. All three cameras are used for the collection of still imagery, but just one is used when videos are collected. During the collection of video imagery the satellite slews to ensure the telescope is continually pointed at the area of interest (AOI).

### Stabilisation and rectification of satellite video imagery

**Fig. 6:**
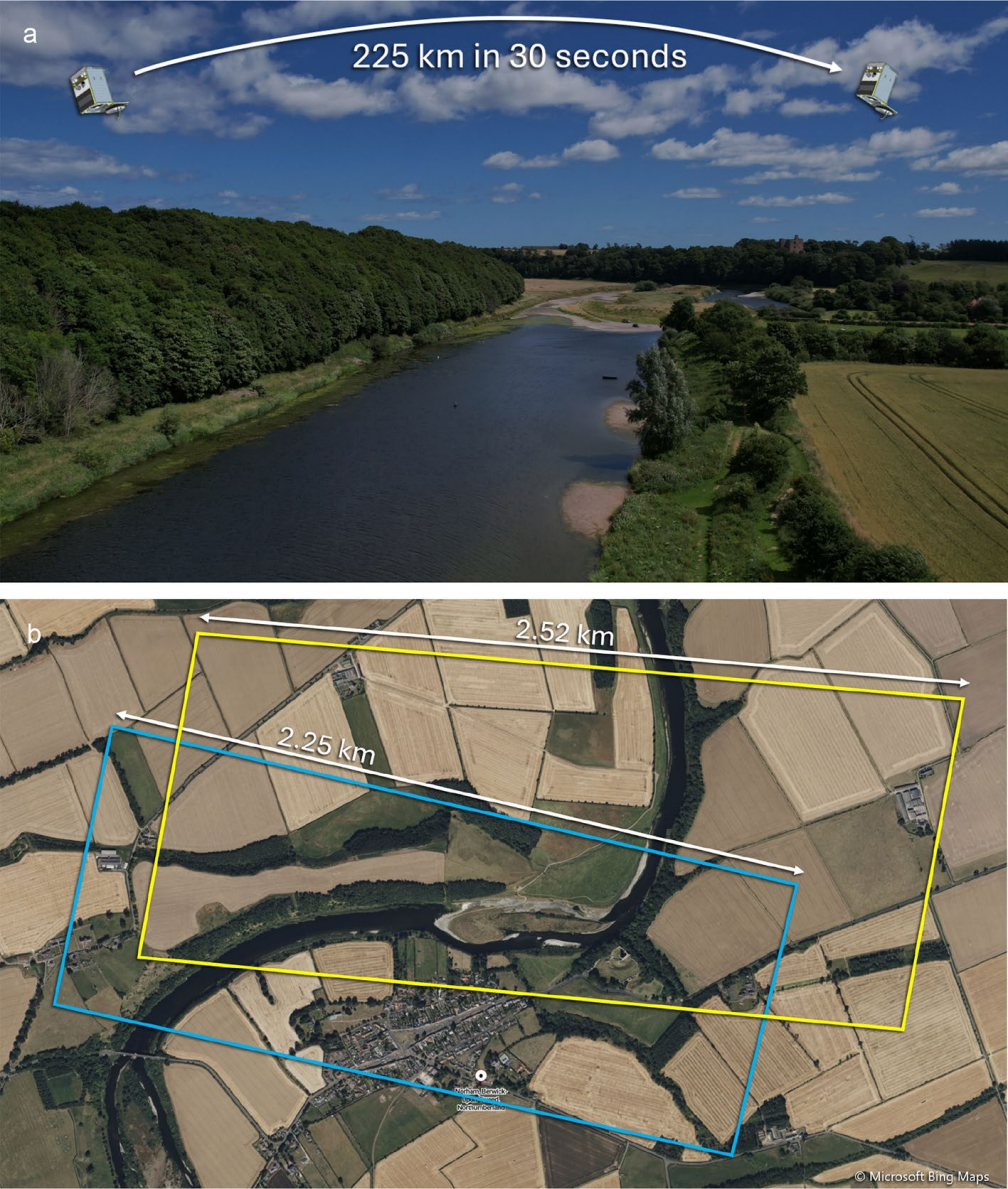
The impact of satellite displacement relative to an area of interest (AOI) during a 30-s satellite video acquisition. (**a**) illustrating the impact of displacement on the viewing angle and range to AOI (picture taken from drone camera for illustration only, not to scale) and (**b**) the impact on the scaling of the imaged scene over the duration of a 30-s video acquisition. The latter is illustrated by overlaying the outline of the first frame (coloured yellow) over that of the last frame (coloured blue) of a 30-s video of the River Tweed at Norham, Scotland. As the satellite position changes, the width of the footprint (or swath) of the imagery on the ground can be seen to change from 2.52 km at the start of the video to 2.25 km at the end. The impact on the imagery of the slightly off-nadir view is evident in the yellow outline which is distorted slightly from the rectangular form of the satellite’s imaging system. Base map imagery:. © Microsoft Corporation, © Maxar Technologies. Image obtained from Bing Maps [accessed July 2025]. Satellite imagery outlines added using Microsoft PowerPoint.

Before water speed analysis can be undertaken, the video imagery acquired by the SkySat satellites requires stabilisation and rectification to address wandering of the scene due to minor instability of the imaging equipment, as well as warping and changes to the Ground Sample Distance caused by the satellite’s rapidly changing position in its orbit (see Fig. 6).

To enable velocimetry analysis, it is essential that all frames of the video used are pre-processed so that they represent the same exact location and spatial extent on the earth’s surface. This was enabled by obtaining satellite videos for precisely defined AOIs where fixed features on the riverbanks could be used to enable very precise rectification and stabilisation of the imagery.

A custom video stabilisation algorithm was developed, which sought to find the perspective transformation between consecutive frames. Integrating frame-to-frame transformations enables the projection of every frame to the coordinate system of the first frame of the given input video. Estimating the parameters of the perspective transformation matrix is done by finding a matrix that minimises geometric errors for multiple pairs of image coordinates of the same geographic location in previous and current frames.

One problem of perspective transformations is the assumption that both sets of keypoints lie on the same plane in the real world. This does not hold well for some areas with significant variations in surface elevation. Here, the stabilisation framework was extended to utilize a user-defined list of polygons that described the regions of interest. Then, keypoint pairs were filtered prior to transformation processing to only consider points that lay inside the AOI.

A critical assumption underlying our approach is the concept of a static environment, a notion particularly challenged by cloud movements, as they shift independently from the ground and satellite. This dynamic behaviour of clouds, when overlooked, led to significant position drifts during stabilization across all keypoints. To address this, we utilized a Semantic Segmentation Network derived from the HalfUnet architecture^33^, incorporating efficient GhostNetV2 modules^34^ as feature extractors to optimize performance. The network was trained on grayscale imagery sourced from the 38-Cloud Dataset^35^, which provides a robust foundation for cloud segmentation tasks due to its diverse satellite imagery samples. Our goal was to accurately detect cloud-covered regions in SkySat Video frames to support water flow speed estimation by stabilizing keypoints in cloud-free areas.

To bolster the network’s generalization across varying spectral conditions, we implemented a novel data augmentation strategy during training. This technique involves randomly generating linear combinations of the Red, Green, Blue, and Near Infrared (NIR) channels. By blending these channels with randomized weights, we simulate a range of atmospheric and illumination scenarios that the network might encounter in real-world satellite data. This augmentation enhances the model’s robustness, enabling it to perform effectively on unseen SkySat Video frames without requiring additional fine-tuning.

The trained network outputs binary segmentation masks that distinguish cloud-covered regions from cloud-free areas. These masks play a critical role in the stabilization process by filtering out keypoints located in cloud-obscured regions. During video frame stabilization, keypoints in areas identified as cloud-covered are excluded, mitigating drift that arises from cloud motion between frames. By focusing solely on keypoints in stable, cloud-free zones, we significantly reduce errors in tracking and improve the reliability of subsequent analyses.

This approach directly contributes to enhancing the accuracy of water flow speed measurements. By ensuring that only stable, visible ground features are tracked across frames, the system minimizes the impact of transient cloud movements, leading to more robust stabilization and thus precise velocity estimates. The integration of an advanced segmentation network, innovative augmentation, and strategic mask application provides a comprehensive solution for processing dynamic satellite video data. Additionally, we employed robust geometric augmentations alongside the CowMask^36^ augmentation. These techniques collectively enhanced the network’s generalization capabilities to such an extent that no further fine tuning was required on SkySat Video frames to produce high-quality binary masks. Consequently, this approach allowed us to selectively focus stabilization efforts on keypoints unaffected by cloud coverage.

### Field data for validation

Locations for satellite video acquisitions were chosen where accurate validation measurements could be made using established methods. Validation sites were also selected for their suitability for the satellite velocimetry technique, which was expected to perform best on water flowing at speeds in excess of 1 m per second, and with a significant degree of surface roughness (Table 1).

**Fig. 7:**
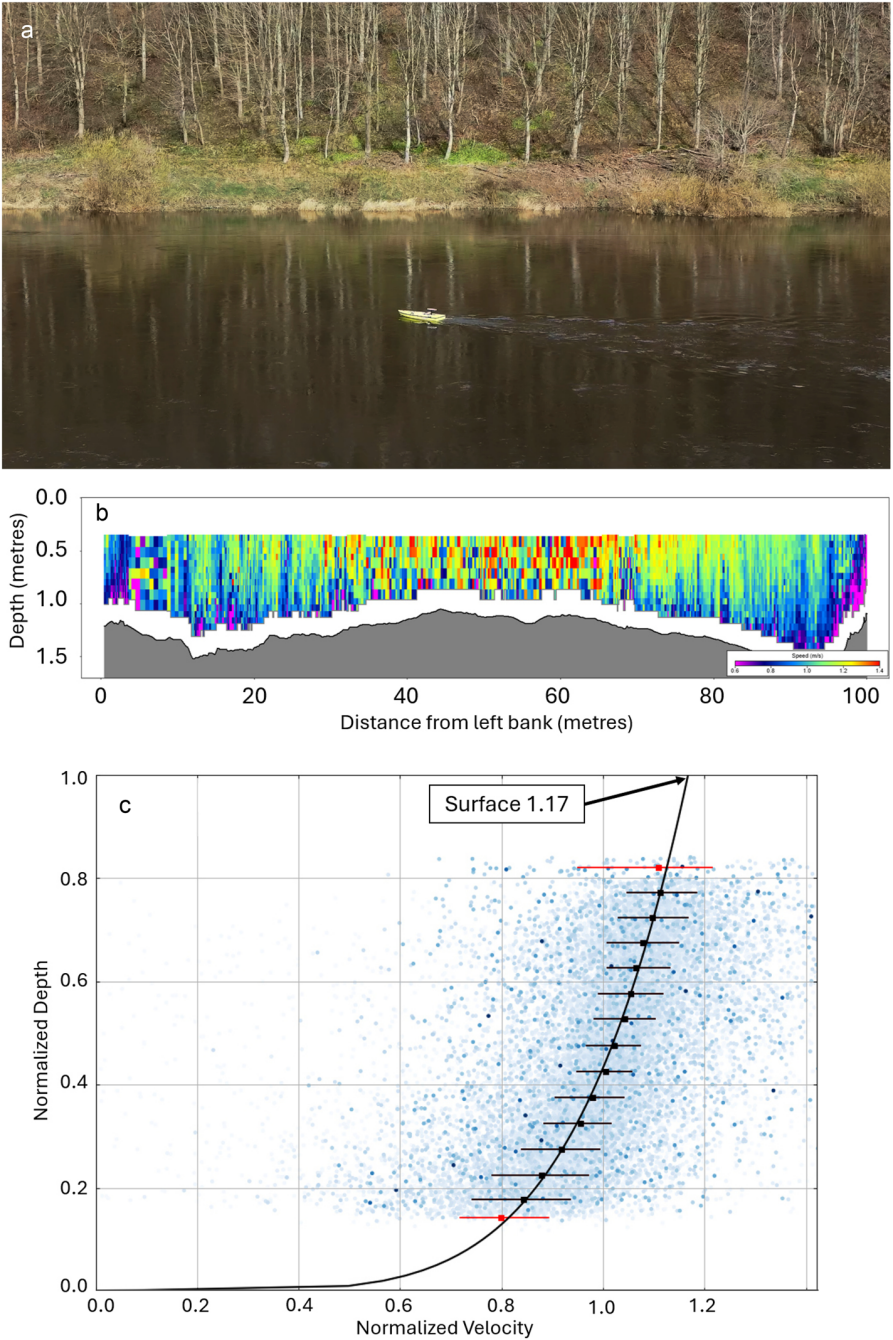
Measuring river discharge using an Acoustic Doppler Current Profiler (ADCP) deployed on the uncrewed ARCboat. (**a**) The ARCboat remote-control boat with onboard Sontek M9 ADCP, deployed on the River Tweed at Norham, Scotland. The boat is controlled by an operator from the riverbank. (**b**) An ADCP river discharge measurement comprises a minimum of four transects of the river perpendicular to the flow direction, in which the ADCP is moved slowly and continuously from one bank to the other. Illustrated here is a single ADCP transect of the River Tweed at Norham, Scotland. Water speeds are represented by colour, with the fastest water close to the surface and towards the centre of the channel shaded red, flowing at around 1.4 m a second. The data appears noisier in the middle of the river as the ADCP has adapted its sampling for the faster water. The detailed riverbed cross-section generated by the ADCP is used for the calculation of discharge with the satellite videos. (**c**) An ADCP discharge profile for the River Tweed at Norham in Scotland, plotted in QRevInt software. Here, the unit discharge (analogous to relative downstream velocity) at the surface is 117% of the mean, indicating that the surface water speed is 17% faster than the mean water speed, resulting in a surface alpha value of 0.855.

Validation comprised an intercomparison of satellite-derived surface water speeds (the FluViSat method) with water speeds derived from aerial camera drones and (where possible) river discharge measurements made with Acoustic Doppler Current Profilers^37^ (ADCPs) deployed with crewed and uncrewed boats (Fig. 7a). Planned validation exercises were not possible at several sites due to low flows and/or problems with cloud cover and these are not covered in this paper. The site at the Falls of Lora in Scotland was included despite proving unsafe for ADCP deployments. As it is a tidal race, where very large volumes of water rush in and out of 30-km long Loch Etive with the rise and fall of the tide over a fjordic sill^38^, the site offered the advantage of a highly predictable timing and scale of very large flows of water, though it also brought challenges associated with rapidly changing tidal currents.

### Surface alpha

Velocity-area discharge calculation techniques based upon cross-sectional area and flow velocity require that the mean downstream velocity in the measured cross-section be determined. The speed of water flow at the free surface of a river is however not generally representative of the mean water speed in the water column or cross-section, due to factors such as bed roughness, water depth and channel geometry^39^. To overcome this problem, a multiplier known as a surface alpha coefficient is used^40^. This multiplier is applied to the observed downstream surface water speed in order to make it representative of the bulk mean downstream velocity in the river channel^41^. The multiplier can be based upon hydraulic theory or may be empirically derived, based upon for example, the analysis of ADCP vertical velocity or discharge profiles and is typically in the range 0.75 to 0.95. As an example, for the River Tweed, the surface water speed was determined to be 117% (i.e. 1.17 times) of the mean water speed, so a surface alpha value of 0.855 was used.

$$\frac{1}{1.17} \approx 0.855$$

For this research, the open source QRevInt ADCP data validation and analysis tool^42^ was used to derive the correct surface alpha value based upon the vertical discharge profile in ADCP data (Fig. 7a & c). For the Burdekin River observation on May 18^th^, when deployment of the ADCP was not possible, the discharge profile and cross-section from the February measurements was used, with an adjustment made to allow for the increased water depth. Where ADCP data was not available to provide discharge profile information and the other important variable, cross-sectional area, discharge was not calculated.

### Drone based velocimetry

To provide the most direct comparison with satellite-derived surface water speeds, where possible aerial camera drones were flown over the waterbodies on the same day as the satellite acquisition (Table 6). The videos acquired were processed using the same procedures in the HydroSTIV software as were applied to the satellite videos (details provided below). Drone videos of 20- or 30-s durations were captured in accordance with the published Australian National Guidelines for drone-based river flow monitoring^43^.

**Table 6:** Dates on which satellite and drone data were acquired.

Site	Satellite video acquisition date	Drone video acquisition date	Simultaneous?	Reason if not simultaneous
Burdekin River at Hydrosite	7/2/22	7/2/22	Yes	
Burdekin River at Hydrosite	8/2/22	8/2/22	Yes	
Burdekin River at Hydrosite	18/5/22	18/5/22	Yes	
Uono at Negoya Bridge	10/5/22	10/5/22	Yes	
River Tweed at Norham	17/3/22	15/3/22	No	Cloud cover prevented satellite acquisition during field trip
Rhine at Rhine Falls	17/10/22	18/08/22	No	Issues with cloud cover and accurate targeting of AOI during and after field trip delayed the satellite video acquisition

**Fig. 8:**
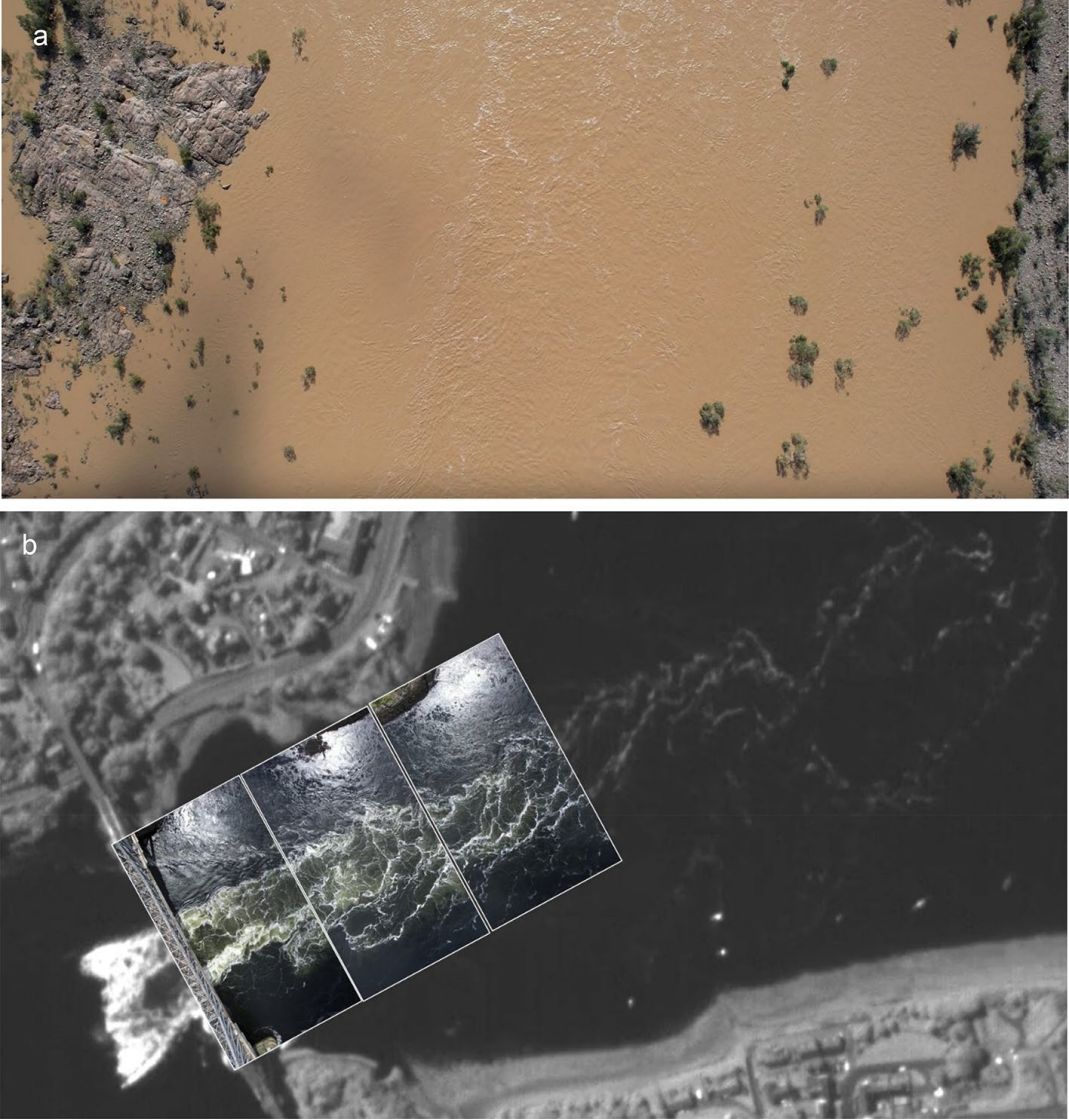
Intercomparison of drone and satellite imagery. (**a**) One frame from a drone video of the Burdekin River, Queensland, Australia, May 18th, 2022. (**b**) Footprint of aerial drone videos overlaid onto a frame of SkySat satellite video at the Falls of Lora. Note, the SkySat video frame has been cropped substantially to show drone videos effectively. Modified (cropped) frame from satellite video imagery obtained by Planet Labs’ SkySat S104 satellite, reproduced with kind permission of Planet Labs PBC. Drone imagery outlines added using Microsoft PowerPoint.

The drones were flown at an altitude that enabled the videos to capture the full width of the waterbody with a nadir view (Fig. 8a). An exception was at The Falls of Lora, Scotland, where the full extent of the water could not fit into the view due to altitude restrictions associated with the nearby Oban Airport (Fig. 2a**)**. Here, videos were shot that captured as much of the flowing water as possible, and the same portion of the satellite videos was analysed to ensure comparability of results (Fig. 2e).

Water speed values derived from aerial drone video imagery are more directly comparable to those obtained from the satellite videos than for example ADCP data because:

- Readings from both represent the speed of flow of the free surface of the waterbody only.
- A very similar, or identical sampling time is used.
- Water speed across the full width of the waterbody is assessed simultaneously.

Water speed information obtained from ADCPs, while extremely informative, is rather less directly comparable for several reasons. Firstly, ADCP systems are unable to measure all the way to the free surface of a river. This is because the ADCP’s acoustic sensors must be submerged a few centimetres below the water’s surface and the physical presence of the instrument and flotation boat in the flowing water can bias results close to the surface. Secondly, discharge measurements with ADCPs are made with the sensor in continuous motion and as a result, any given location in each transect is only sampled for a very short period. The impact of this is apparent in the plots in Fig. 1b & c, where a greater degree of variation can be seen in the ADCP velocity values compared to those derived from aerial drones.

### Determination of water surface speed

**Fig. 9:**
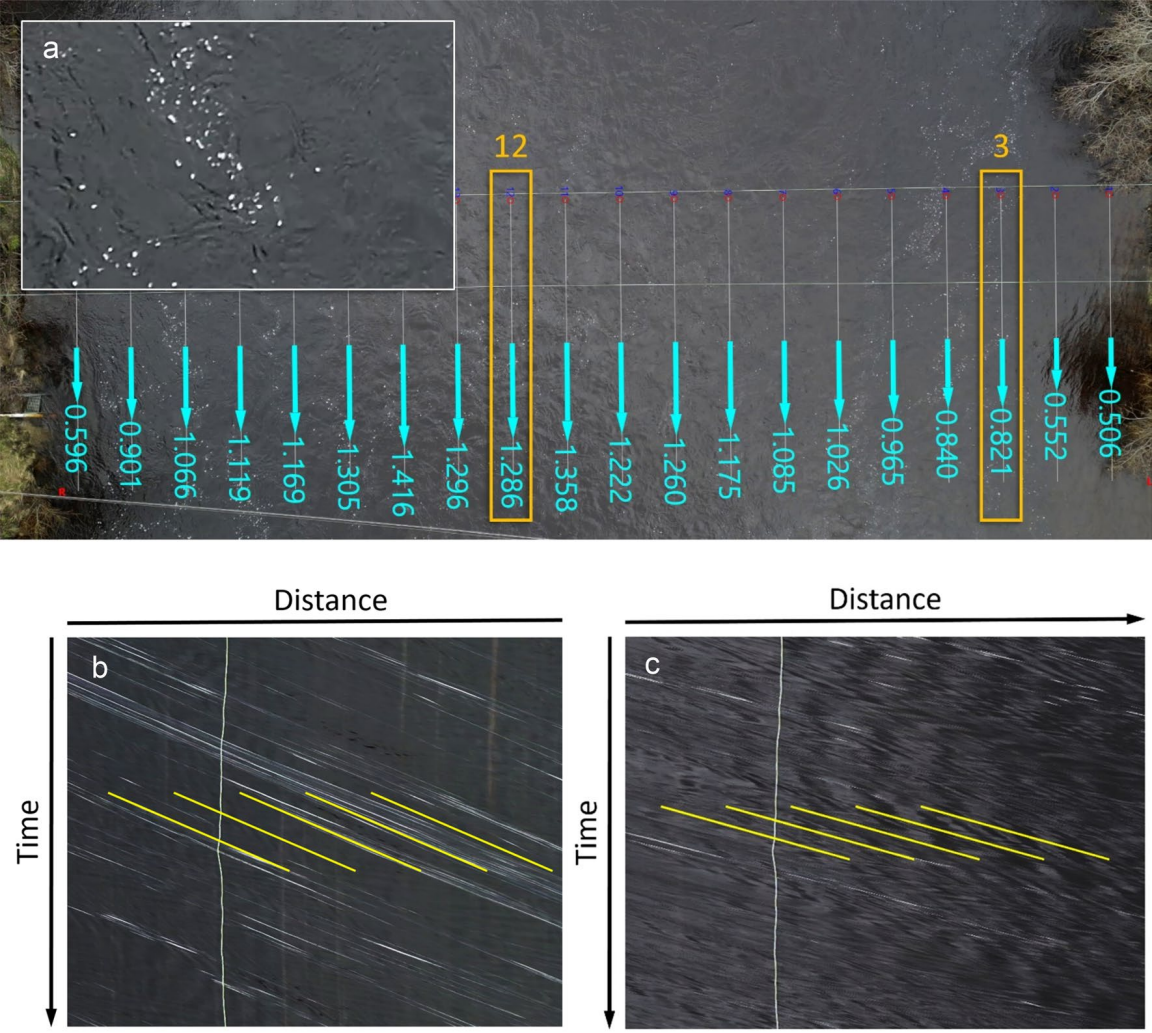
Space Time Imaging Velocimetry: How it works. (**a**) Aerial view of the River Garry at Killiecrankie in Scotland (chosen because the type and distribution of tracers was ideal for video velocimetry), showing velocity vectors developed through STIV analysis of a drone video. Inset shows roughness features and patterns of white foam on water’s surface that are tracked long the faint white ‘search lines’ that can be seen aligned with the primary direction of flow of the river. (**b**) Space Time image for search line 3 (highlighted in orange box in 9a) from HydroSTIV software. Individual frames of the video are ‘stacked’ down the screen for each search line. The diagonal white streaks illustrate the displacement along the lines of brightness features (in this case, the surface foam) advecting with the flow. Inclined yellow lines show mean angle from STIV analysis. The vertical white line running through the STIV images is a public utility cable that can be seen in image (**a**) spanning the river and intersecting the search lines. This does not bias results. (**c**) Space Time image for search line 12, showing faster water speed (yellow analysis lines are closer to the horizontal), and the presence of surface roughness features that are advecting with the flow in addition to the foam features.

After stabilisation and rectification, water surface flow speed was derived from the SkySat videos using the commercially available HydroSTIV velocimetry software application which employs a very robust processing technique known as Space Time Imaging Velocimetry, or STIV^25,42^. The STIV method is based upon tracking variations in brightness caused by the movement of visible features (tracers) on the water’s free surface along user-defined ‘search lines’ which are aligned with the flow direction (see Fig. 9). For each search line, variations in brightness are plotted for every frame of the video. The resultant linear plots of brightness are stacked one below the other to form a single composite ‘Space Time’ image, with each horizontal line representing one frame of the video.

If a tracer moves in a downstream direction, its brightness signature is displaced slightly further to the right in each successive row of the space time image. This results in the appearance of diagonal streaks in the composite image, as the tracer moves through space (i.e. downriver) and time (through the duration of the video). Since many tracers may pass along each search line over the course of a 30 s video, multiple broadly parallel diagonal streaks will result. The steepness of these diagonal lines reveals the speed of motion of the tracer being tracked, and hence the water’s surface speed. This simple but robust methodology enables STIV software to accurately track the downriver motion of all visible features advecting with the flow on the water’s surface, including debris, foam and turbulent roughness features^44^. The STIV processing methodology also proved very robust in dealing with the sometimes relatively indistinct surface feature signatures that result from the 400–450 km range to the target in satellite videos. The same HydroSTIV software and methods were used to process satellite and drone sourced videos.

The HydroSTIV software provides a very simple and robust method of determining water speed from the video files, but occasionally the results can be compromised by unusual illumination or water surface patterns. In this case, the software provides the user with a tool to re-analyse the space–time images and make manual adjustments. For drone-derived videos, it is occasionally necessary to make manual adjustments for areas of the water where visible tracers are scarce, or surface roughness patterns are misinterpreted by the velocimetry algorithms.

For the satellite videos, while results obtained in the stronger flows towards the centre of the river channels tended to be good, manual controls were sometimes used to refine velocity assessments close to the riverbanks where the optical signal was less strong.

Due to the relatively large pixel sizes of the satellite imagery (typically in the range of 0.80–0.95 m), search lines used for analysis of water speed in the satellite videos were longer than for drone videos, in order to include sufficient pixels to enable robust analysis.

## Calculation of discharge

To calculate discharge from satellite observations, the same well-proven velocity-area method^31^ was applied as is standard practice for drone-based measurements. In this method, river discharge is the product of the river’s cross-sectional area multiplied by the mean speed of the water flowing through that cross-section^45^. Camera-based surface-velocity river discharge measurements differ from the majority of traditional velocity-area methods in that only the speed of flow of the free surface of the waterbody is measured directly as part of the observation. For surface velocity measurements, the mean water speed is determined by multiplying the velocity observed at the free surface by the surface alpha value derived from the ADCP data (as previously described). Since neither the drone nor the satellite has any means of measuring the water’s depth, the river’s cross-sectional area was also derived from the ADCP measurements. For this reason, at sites where ADCP measurements could not be made, discharge was not calculated.

## Data Availability

The drone imagery datasets used for validation of satellite observations in the current study are available through the Natural Environment Research Council Environmental Information Data Centre via 10.5285/d62347ba-0173–4857-b3fc-b7238988f0d8. Raw ADCP datasets created and analysed during the current study can be made available by request to the corresponding author at the UK Centre for Ecology and Hydrology: www.ceh.ac.uk. The satellite videos were used under license for the current study and are available upon reasonable request from the corresponding author’s institute, the UK Centre for Ecology and Hydrology: www.ceh.ac.uk, which has been granted limited permission from Planet Labs PBC to share the imagery.
